# Estimates of array and pool-construction variance for planning efficient DNA-pooling genome wide association studies

**DOI:** 10.1186/1755-8794-4-81

**Published:** 2011-11-28

**Authors:** Madalene A Earp, Maziar Rahmani, Kevin Chew, Angela Brooks-Wilson

**Affiliations:** 1 Canada's Michael Smith Genome Sciences Centre, BC Cancer Agency, Vancouver, BC, Canada; 2 Department of Medical Genetics, University of British Columbia, Vancouver, BC, Canada; 3 Department of Biomedical Physiology and Kinesiology, Simon Fraser University, Burnaby, BC, Canada

## Abstract

**Background:**

Until recently, genome-wide association studies (GWAS) have been restricted to research groups with the budget necessary to genotype hundreds, if not thousands, of samples. Replacing individual genotyping with genotyping of DNA pools in Phase I of a GWAS has proven successful, and dramatically altered the financial feasibility of this approach. When conducting a pool-based GWAS, how well SNP allele frequency is estimated from a DNA pool will influence a study's power to detect associations. Here we address how to control the variance in allele frequency estimation when DNAs are pooled, and how to plan and conduct the most efficient well-powered pool-based GWAS.

**Methods:**

By examining the variation in allele frequency estimation on SNP arrays between and within DNA pools we determine how array variance [var(e_array_)] and pool-construction variance [var(e_construction_)] contribute to the total variance of allele frequency estimation. This information is useful in deciding whether replicate arrays or replicate pools are most useful in reducing variance. Our analysis is based on 27 DNA pools ranging in size from 74 to 446 individual samples, genotyped on a collective total of 128 Illumina beadarrays: 24 1M-Single, 32 1M-Duo, and 72 660-Quad.

**Results:**

For all three Illumina SNP array types our estimates of var(e_array_) were similar, between 3-4 × 10^-4 ^for normalized data. Var(e_construction_) accounted for between 20-40% of pooling variance across 27 pools in normalized data.

**Conclusions:**

We conclude that relative to var(e_array_), var(e_construction_) is of less importance in reducing the variance in allele frequency estimation from DNA pools; however, our data suggests that on average it may be more important than previously thought. We have prepared a simple online tool, PoolingPlanner (available at http://www.kchew.ca/PoolingPlanner/), which calculates the effective sample size (ESS) of a DNA pool given a range of replicate array values. ESS can be used in a power calculator to perform pool-adjusted calculations. This allows one to quickly calculate the loss of power associated with a pooling experiment to make an informed decision on whether a pool-based GWAS is worth pursuing.

## Background

Genome-wide association studies (GWAS) have been used to examine over 200 diseases and traits, and identified over 4000 single nucleotide polymorphisms (SNPs) associated with these traits, as listed in the *Catalog of Published Genome-Wide Association Studies *[1]. In many cases, GWAS have revealed previously unsuspected molecular mechanisms of disease, highlighting the value of this hypothesis-free approach [reviewed in [2,3]]. Unfortunately, GWAS are very costly due to the price of genotyping thousands of individual DNA samples on high-density SNP arrays. Consequently, GWAS have only been feasible for research groups with the necessary budget, studying well-funded diseases or traits. A simple strategy to drastically reduce cost is to replace individual genotyping in Phase I of a GWAS with genotyping of DNA pools. DNA pools yield estimated allele frequencies rather than observed genotypes; hence, this step has been called allelotyping [4]. Several studies have provided proof of principle for the pooling strategy, using it to re-discover known disease-variant associations of moderate to large effect size for a fraction of the cost of conventional GWAS [5,4]. To date, more than twenty pooled-based GWAS have been published, many reporting genome-wide significant associations for diseases and traits such as follicular lymphoma, otosclerosis, multiple sclerosis, Alzheimer's disease, melanoma, psoriasis, and skin colour [6-12]. Depending on the number of samples being pooled, the cost reduction in Phase I can easily reach 100 fold. Consider, if a SNP array costs $250 and there are 2000 cases and 2000 controls to genotype, a million dollars is required for Phase I individual genotyping alone. Conversely, the pool-based experiment using 12 replicate arrays on two pools (case and control) would be $6000, or 0.6% of the cost. Simply put, a pooling GWAS is feasible for most grant budgets, while an individual genotyping GWAS is not. The criticism of pool-based GWAS is that they have reduced power relative to conventional GWAS because of errors introduced by estimating allele frequency from DNA pools rather than individual genotyping data. While it is true that pool-based GWAS forfeit some power, these losses can be estimated, are often less than expected, and may not change the associations discovered. Although array costs will continue to drop and conventional GWAS will become more feasible, the potential savings associated with the pooling approach will scale in proportion, leaving more funds for subsequent replication, fine-mapping, and sequencing of associated genomic regions. For diseases or traits with unknown biology or genetic involvement, a pooling GWAS represents an economical way to test for associations with moderate odds ratios. In addition, work using DNA extracted from pooled whole blood suggests that a large time-savings (50-100 fold) may also be possible, presenting the possibility of an incredibly fast (<1 month) and economical experiment [5]. For a comprehensive introduction and review of DNA pooling readers are directed to Sham et al. 2002 and Pearson et al. 2007 [13,4], and for a set of best practices for any GWAS to Pearson & Manolio, 2008 [14].

We know that in the process of estimating allele frequencies from DNA pools we introduce error, and these must be taken into consideration to plan an adequately powered experiment or to appropriately calculate association statistics [15,16]. With respect to doing this, the most important consideration is the pooling variance [17]; the variance in the errors arising from estimating allele frequency from a DNA pool. Pooling variance is the sum of many sources of variation, including in particular, array variance and pool construction variance. Array variance can be attributed to those errors arising from estimating allele frequency from a DNA pool on an SNP array [17,18]. Pool construction variance can be attributed to those errors arising from the physical creation of a DNA pool. As pooling variance increases, the ability of a pool-based GWAS to detect odds ratios similar to those detectable by conventional GWAS decreases. In this report we assume pooling variance is the sum of array variance and pool-construction variance and attempt to determine which makes the greater contribution to the pooling variance. This is relevant to determining how best to design a pool-based GWAS and how to allocate resources, for example, replicate arrays can be used to reduce array variance and/or pools can be constructed in replicate to control pool construction variance.

Here we partition and estimate variance components using the approach described by MacGregor [17], which examines variation in allele frequency measurements between and within DNA pools. Briefly, within-pool variation is that observed between two arrays used to allelotype the same DNA pool (i.e. replicate arrays), and is an estimate of array variance. Between-pool variation is that observed between two arrays used to allelotype two different DNA pools, and is an estimate of pooling variance. Estimates of array variance and pooling variance are used to calculate pool construction variance by subtraction [17]. Using this approach in an analysis of two DNA pools allelotyped on twelve Affymetrix Genechip HindIII arrays (6 arrays per pool) MacGregor [7] found that approximately 87.5% of pooling variation could be attributed to the arrays, leaving 12.5% to pool-construction [17]. It was noted, however, that more data sets would be necessary to determine the variability in these estimates. Here we inspect 27 DNA pools allelotyped on a total of 128 Illumina arrays, including the Human1M Single (1M-Single), Human1M Duo (1M-Duo), and HumanHap660 Quad (660-Quad) arrays, allowing us to better address the question of what values array variance and pool-construction variance are likely to take. In addition, we perform our analysis on normalized array data and raw array data to examine how normalization affects pooling variance estimates.

In the first part of this study we establish values for array variance and pool-construction variance. In the second part, we use these estimates to calculate the effective sample size (ESS) of a DNA pool (where ESS is the equivalent number of samples that would need to be individually genotyped to give a similar result) [19]. We also present a simple online tool, PoolingPlanner, which uses our empirical variance estimates as default values to calculate the effective sample size (ESS) of a DNA pool given a range of replicate array values (available at http://www.kchew.ca/PoolingPlanner/). PoolingPlanner also accepts user-supplied values for variance estimates. ESS can then be used in one of the available power calculators, such as CaTS [20], or Quanto [21], to perform pool-adjusted power calculations [4]. PoolingPlanner is intended to help researchers quickly calculate the loss of power associated with a particular pooling experiment, which is a first step in making on informed decision on whether a pool-based GWAS is worth pursuing.

## Methods

### Data

Our analysis is based on 27 DNA pools ranging in size from 74 to 446 individual samples. These were allelotyped on a collective total of 128 Illumina beadarrays: 24 1M-Single, 32 1M-Duo, and 72 660-Quad. Our dataset comprises four batches of genotyping (details given in Additional File 1, **Table S1**), which correspond to four ongoing pool-based GWAS that have not yet been published. Each of these studies was approved by the joint Clinical Research Ethics Board of the British Columbia Cancer Agency and the University of British Columbia. All subjects gave written informed consent.

Genomic DNA was extracted from peripheral venous blood collected between 2001 and 2008 by different laboratories using different methods. DNA samples were diluted to 50-100 ng/uL and then quantified in duplicate by fluorometry using PicoGreen™(Molecular Probes, Eugene, OR, US). Pools were constructed by combining 200 ng of each sample DNA by manual pipetting. Pools were assayed (allelotyped) at the Centre for Applied Genomics at Sick Children's Hospital in Toronto."

SNP allele frequency in DNA pools was estimated using Illumina's beadarrays, where on average each SNP is estimated by 16-18 "bead" observations per array (oligonucleotide probes are designed to assay a SNP and attached to beads, where individual beads are coated with one probe type and interrogate one site in the genome) [22]. **Equation 1 **was used in the calculation of each SNP allele frequency:

(1) $${\hat{p}}_{i=1...n}=\frac{1}{n}\overset{n}{\underset{i=1}{\sum }}\frac{G_{i}}{G_{i}+R_{i}}$$

where G*_i _*and R*_i _*are the green and red fluorescence intensity for the *i*th bead assaying a given SNP. The two colours correspond to the two alleles of the SNP, and *n *is the number of beads assaying a given SNP, typically 16-18. Illumina beadarrays are manufactured such that there are multiple strips on each array [22], and our preliminary analysis revealed that unique groups of SNPs are consistently on only a subset of strips. From our previous experience, and that of others [18], it was known that the average relative intensity of the red and green channels could differ dramatically between strips and between arrays. To prevent these manufacturing and/or assaying properties from biasing allele frequency estimation, a simple normalization was performed. Each array was normalized on a strip-by-strip basis by adjusting the red channel intensity to give a mean strip-wide allele frequency estimate of 0.5 [18]. To examine the effect of this normalization on the variance terms estimated, the analyses presented in this paper are performed on both normalized and raw Illumina array data.

### Statistical Analysis

Our purpose is to calculate empirical estimates of pooling variance and array variance, and then to estimate pool construction variance by subtraction. Pooling variance and array variance are both estimated by calculating allele frequency differences across two paired (by SNP, for all SNPs on the array) arrays [17]. The two arrays used in the comparison will dictate whether an estimate of array or pooling variance is generated. For example, to calculate array variance, let allele frequency estimates on arrays *x *used to allelotype DNA pool *a *be:

$${\tilde{p}}_{ax}={\hat{p}}_{a}+e_{array\text{\_}x}$$

where is the true allele frequency for those samples in DNA pool *a*, and e_array_x _is the error associated with estimating the allele frequency from a DNA pool [15]. Then, the variance of the allele frequency difference on two replicate arrays (*x *= 1, 2) is [17]:

$$\begin{array}{ll} \text{var}\;\left({\tilde{p}}_{a1}-{\tilde{p}}_{a2}\right) & =\text{var}\;\left({\hat{p}}_{a}+e_{array\text{\_}1}-{\hat{p}}_{a}-e_{array\text{\_}2}\right)\;\\ & =\text{var}\;\left(e_{array\text{\_}1}-e_{array\text{\_}2}\right)\;\\ & =2\text{var}\;\left(e_{array}\right)\;\\ & \; \end{array}$$

This yields an estimate of array variance:

$$\text{var}\;\left(e_{array}\right)=\text{var}\;\left({\tilde{p}}_{a1}-{\tilde{p}}_{a2}\right)∕2$$

where $\text{var}\;\left({\tilde{p}}_{a1}-{\tilde{p}}_{a2}\right)$ is calculated as the average of the squared allele frequency differences for all SNPs, *i *(*i *= 1...n), on arrays 1 and 2:

$$\text{var}\;\left({\tilde{p}}_{a1}-{\tilde{p}}_{a2}\right)=\frac{1}{n-2}\overset{n}{\underset{i=1}{\sum }}{\left({\tilde{p}}_{a1,i}-{\tilde{p}}_{a2,i}\right)}^{2}$$

*Var*(*e_array_*) is assumed constant for all SNPs. If more than two replicate arrays are used to allelotype a given DNA pool, multiple array comparisons are possible, and the best estimate of var(*e_array_*) is the average of all possible pairings [17].

If arrays 1 and 2 interrogate two different DNA pools, an estimate of pooling variance can be obtained. When two DNA pools (*a, b*) are constructed from identical samples (i.e replicate pool construction),

$$\text{var}\;\left({\tilde{p}}_{a1}-{\tilde{p}}_{b2}\right)=2\text{var}\;\left(e_{array}\right)+2\text{var}\;\left(e_{construction}\right)$$

where var(*e_construction_*) is the variance in the pool construction errors, which are assumed to be constant for all SNPs. Thus, an estimate of pooling variance, var(*e_pooling-_*_1_) is [17]:

$$\text{var}\;\left(e_{pooling-1}\right)=\text{var}\;\left({\tilde{p}}_{a1}-{\tilde{p}}_{b2}\right)∕2$$

where "pooling-1" is used to indicate that this estimate of pooling variance is based on the comparison of arrays that allelotype two replicate DNA pools. As before, if more than two replicate arrays are used to allelotype a given DNA pool, multiple array comparisons are possible, and the best estimate of var(*e_pooling-_*_1_) is the average of all possible pairings [17].

When DNA pools *a *and *b *are constructed from non-identical samples (ex. a case and control pool), an alternative estimate of pooling variance is var(*e_pooling-_*_2_)[15,17]:

$$\text{var}\;\left(e_{pooling-2}\right)=\left[\text{var}\;\left({\tilde{p}}_{a1}-{\tilde{p}}_{b2}\right)-{Ṽ}_{a1,b2}\right]∕2$$

Here $\text{var}\;\left({\tilde{p}}_{a1}-{\tilde{p}}_{b2}\right)$ is calculated as the average of the squared allele frequency difference minus a random binomial sampling variance term, ${Ṽ}_{a1,b2}$, for all SNPs, *i *(*i *= 1...n), on arrays 1 and 2:

$$\mathrm{var}(e_{pooling-2})=\frac{1}{n-2}{\sum_{i=1}^{n}[{\left({\tilde{p}}_{a1,i}-{\tilde{p}}_{b2,i}\right)}^{2}-{\tilde{V}}_{a1,b2,i}}_{}]/2$$

${Ṽ}_{a1,b2}$ is calculated using the usual equation for binomial sampling variance:

$${Ṽ}_{a1,b1,i}=p_{a1,i}\left(1-p_{a1,i}\right)∕N_{a1}-p_{b2,i}\left(1-p_{b2,i}\right)∕N_{b2}$$

The random binomial sampling variance terms accounts for the additional component of variation arising from the comparison of non-identical pools. It is assumed that the two DNA pools are constructed from samples drawn from the same population, and although in fact it is often a case and control being compared (where we specifically look for differences in allele frequency), for most SNPs on an array this is a valid assumption [15].

**Figure 1:**
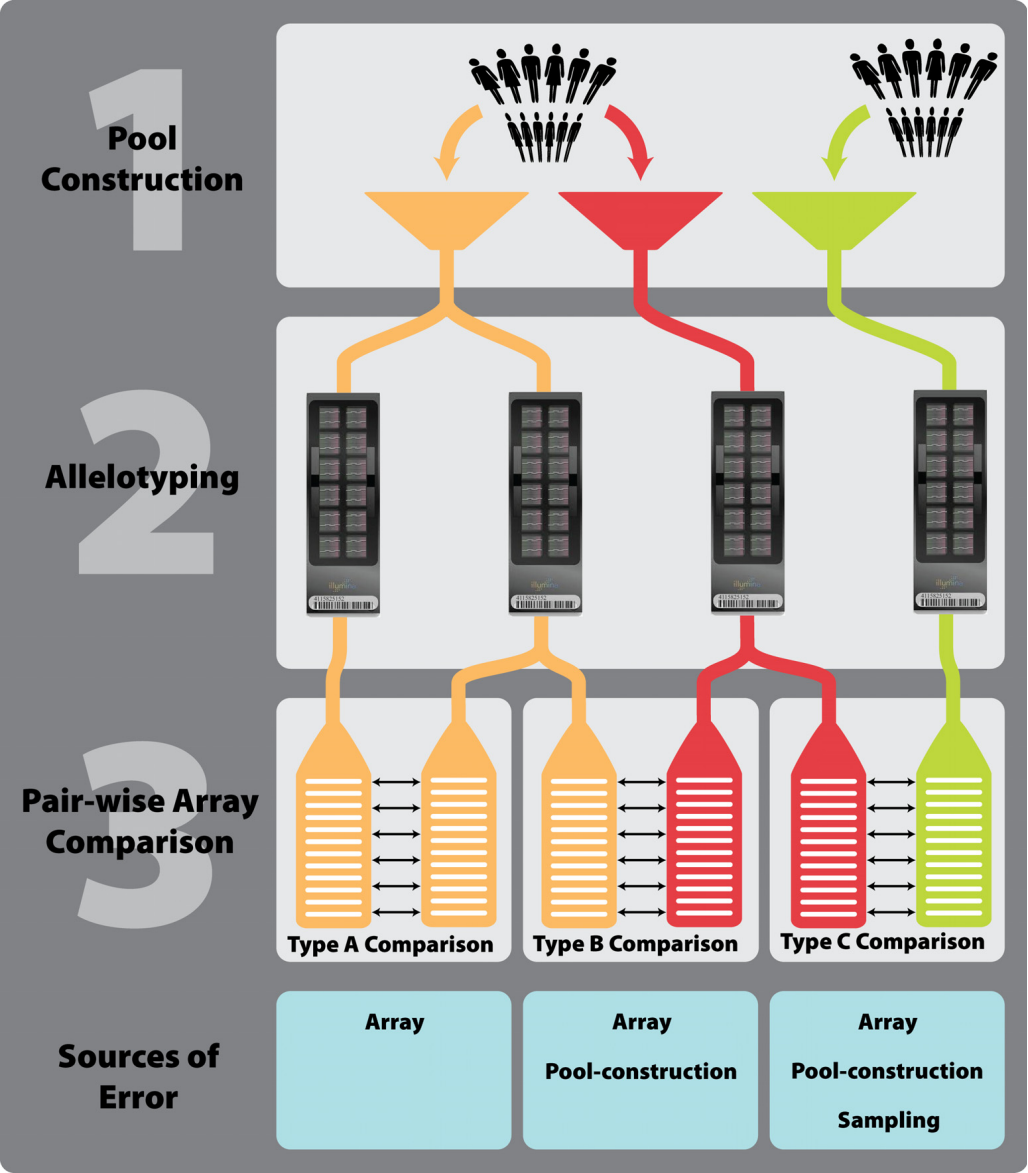
**Overview of the pair-wise array comparison's performed in this study**. *Step 1 *depicts the construction of three DNA pools. The first two pools (orange and red) are constructed using the same DNA samples and are pool-construction replicates. The third pool (green) is constructed using difference DNA samples. *Step 2 *indicates allelotyping on Illumina SNP arrays, where the two arrays allelotyping the orange pool are array replicate. *Step 3 *shows the three types of pair-wise SNP array comparisons that can be made, along with the sources of error that account for differences in allele frequency estimates on the paired arrays. For Type A comparisons, the arrays being compared were used to allelotype the exact same DNA pool; hence, the only source of variation is the array. For Type B comparisons, the arrays paired were used to allelotype independently constructed but identical pools; thus, variation may arise due to the array and the pool-construction process. For Type C comparisons, the arrays paired were used to allelotype completely independent DNA pools, and variation may be due to the array, pool-construction, or binomial sampling (assuming both pools are independent samples from a single population).

Figure 1 visually summarizes the three types of pair-wise arrays comparisons used in this report, including the sources of error in each comparison. When comparing arrays used to allelotype the same DNA pool (henceforth referred to as 'Type A' comparisons), the variation observed can only arise due to the arrays, giving an estimate of array variance. When comparing arrays used to allelotype replicate DNA pools (henceforth referred to as 'Type B' comparisons), the variation observed is due to the arrays and pool-construction, giving a direct estimate of pooling variance. Pool-construction variance is then calculated by subtracting the array variance (Type A) from the pooling variance (Type B). If replicate DNA pools have not been constructed, as is the case for many of the pools in our data set, we are still able to estimate the pooling variance by comparing non-identical pools (henceforth referred to as 'Type C' comparison) and account for the additional binomial sampling variance term that arises in this case. Pool-construction variance is then calculated by subtracting Type A values from Type C values.

A number of assumptions are made in this analysis. We assume that the array variance is comparable across the DNA pools in an experiment, and that the average array variance is the best estimate. For arrays with larger than average array variance, perhaps caused greater variation in PCR amplification steps and/or measurement of allele frequency (detection of red and green fluorescence), array variance will be underestimated; arrays with smaller than average array variance will be overestimated. It is known that SNPs with smaller minor allele frequencies are estimates with a greater margin of error, i.e. var(e_array_) is not constant for all SNPs. For SNP with a small minor allele frequency, average array variance will underestimate the array variance. We also assume that the pooling variance is constant across all SNPs, and that unequal amplification and/or hybridization of alleles (A or B) will have a negligible effect on results. Because our analysis is based upon contrasting array data from two DNA pools, the effects of unequal hybridization should largely cancel out [15,18].

### PoolingPlanner Theory

In choosing to conduct a pool-based GWAS, one accepts a loss in power relative to a conventional GWAS. How much power is lost can be expressed in terms of the effective sample size (N*) resulting from pooling *N *individuals [4]. PoolingPlanner uses an estimate of var(e_pooling_) to calculate the effective sample size of a DNA pool. N* and var(e_pooling_) are related through two expressions for relative sample size (RSS) [defined in 19]:

(2) $$RSS=\frac{N*}{N}$$

(3) $$RSS=\frac{V_{s}}{\left(V_{s}+\text{var}\;\left(e_{pooling}\right)\right)}$$

In one, the RSS of a DNA pool is expressed as the ratio of effective sample size to the actual sample size (*N*). In two, it is expressed as the fraction of the total variance, (V_s _+ var(e_pooling_)), explained by the binomial sampling variance, V_s_. V_s _is calculated as *p*(1-*p*)/2N, where *p *is the average minor allele frequency on the array, and N is number of individuals contributing to the DNA pool. If DNA pools have been constructed in replicate we let var(e_pooling_)= var(e_pooling-1_), otherwise we let var(e_pooling_)= var(e_pooling-2_). The two equations for RSS can then be equated and solved for N*. It is worth noting that because our calculation of RSS relies on our empirical estimates of var(e_pooling_) (Equation 2), estimates which are based on contrasting allele frequencies in two DNA pools, the effects of unequal hybridization, which would typically thwart a direct comparison of a pooling-based and conventional genotyping experiment, cancels out (15, 18).

Replicate arrays can be used to reduce var(e_pooling_) by a factor of 1/*k*, where *k *is the number of replicate arrays [4]. In making var(e_pooling_) smaller the RSS and N* become larger. Effective sample size can then be used with one of the available power calculators, for example CaTS [20] or Quanto [21] to perform pool-adjusted power calculations [4]. PoolingPlanner is intended to help first time users plan a DNA pooling experiment, and our empirical estimates of array variance and pool construction variance are supplied as the default setting for the program for this reason. Users with their own estimates of variances can provide these to the program as well. PoolingPlanner is available at http://www.kchew.ca/PoolingPlanner/).

## Results

In our analyses we encountered beads with negative intensity values in the red, green, or both channels. The number of negative beads varied by strip and typically affected 1-10% beads, a pattern consistently seen across all arrays. This can occur due to local background intensity removal at the point of image processing [23]. These beads were removed from our variance calculations. Furthermore, beads with zero in both the red and green channels were considered failed beads and also dropped from our analysis. There were typically fewer than 100 of these per strip. Finally, SNPs having fewer than four bead observations were excluded. The rationale for this was that SNPs having fewer than four beads observation would have poorly estimated allele frequency.

### Array Variance or var(e_array_): Type A comparisons

**Figure 2:**
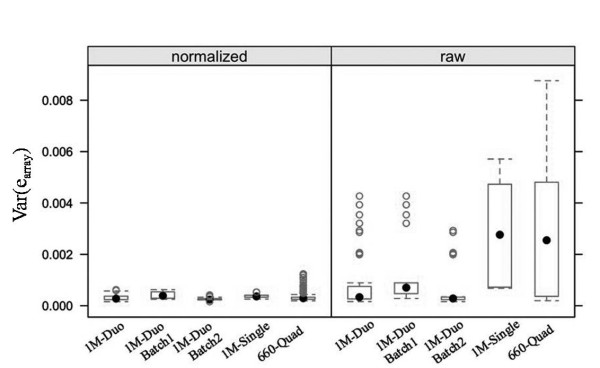
**Box plots of array variance for three Illumina array types**. Box plots of var(e_array(x,y)_) for Illumina 1M-Duo, 1M-Single, and 660-Quad arrays for normalized and raw data. The 1M-Duo arrays were genotyped in two batches and are plotted stratified by batch ("1M-Duo-Batch 1", "1M-Duo-Batch 2"), as well as by array type "1M-Duo". The number of var(e_array_) estimates for each array type is: 1M-Duo, n = 45; 1M-Duo-Batch 1, n = 18; 1M-Duo-Batch 2, n = 27; 1M-Single, n = 11; 660-Quad, n = 360. Box plot whiskers are plotted at the lowest datum within 1.5 the interquartile range of the lower quartile, and the highest datum within 1.5 the interquartile range of the upper quartile.

We estimate array variance by comparing replicate arrays, Type A comparison in Figure 1, for three types of Illumina beadarrays, the 1M-Single, the 1M-Duo, and the 660-Quad. The results for normalized and raw data are given in Table 1, and box plots in Figure 2 provide a visual summary of the estimates. Clearly normalization dramatically reduces the range of observed array variance estimates for all array types. As well, normalization reduced the mean array variance estimate approximately 2.5-fold for the 1M-Duo arrays and approximately 8-fold for the 1M-Single and 660-Quad arrays. For normalized data most estimates of array variance, regardless of array type, fell between 2.5 × 10^-4 ^and 5.0 × 10^-4^.

**Table 1:** Estimates of array variance, var(e_array_), for three Illumina arrays types for normalized and raw data.

	1M-Single	1M-Duo	660-Quad
***Normalized data *Var(e_array_) (Range)**	3.8 × 10^-4^ (2.2 × 10^-4 ^- 6.6 × 10^-4^)	3.2 × 10^-4^ (1.6 × 10^-4 ^- 6.3 × 10^-4^)	3.3 × 10^-4^ (2.5 × 10^-4 ^- 4.9 × 10^-4^)

***Raw data *Var(e_array_) (Range)**	2.9 × 10^-3^ (3.0 × 10^-4 ^- 9.2 × 10^-3^)	9.0 × 10^-4^ (1.7 × 10^-4 ^- 4.3 × 10^-3^)	2.7 × 10^-3^ (2.0 × 10^-3 ^- 3.0 × 10^-3^)

**Number of pools**	12	8	7

**Number of comparisons, var(e_array_)^(1)^**	12	45^(2)^	360

**Number of arrays** **(arrays/pool)**	24 (2/pool)	32 (4/pool)	72 (6 or 12/pool)

^1^Each paired array comparison is treated as an independent estimate of array variance, the average of which is reported in this table.

^2^One array, in all 3 comparisons in which it was involved, produced extreme outlier var(e_array_) values and was removed from all analysis; hence, there are 45 instead of 48 var(e_array_) for the 1M-Duo arrays.

**Figure 3:**
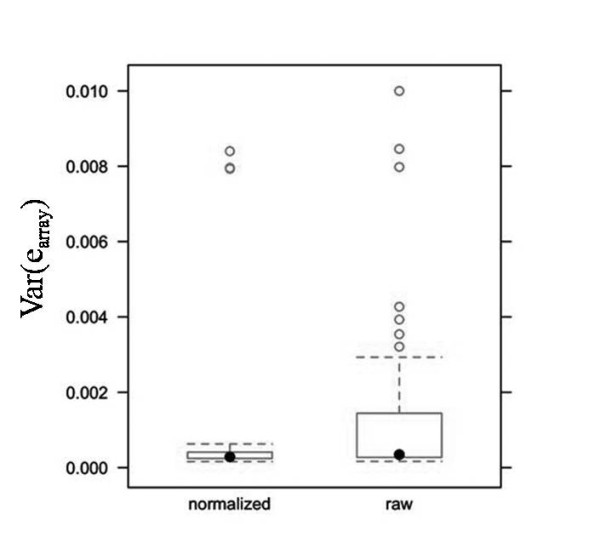
**Box plots of array variance for Illumina 1M-Duo arrays highlighting extreme outliers**. Box plots of var(e_array_) estimates (n = 48) for the 1M-Duo arrays (Batch 1 and 2 combined) highlighting the three extreme outlier estimates in both normalized and raw data, all attributable to one array. This array was determined faulty (see discussion) and removed from all analyses. Box plot whiskers are plotted at the lowest datum within 1.5 the interquartile range of the lower quartile, and the highest datum within 1.5 the interquartile range of the upper quartile.

For the 1M-Single arrays 12 DNA pools were allelotyped using 24 arrays (2 arrays per pool), yielding 12 estimates of array variance, the mean of which was 3.8 × 10^-4 ^(normalized) and 2.9 × 10^-3 ^(raw data), see Table 1. For the 1M-Duo array 8 DNA pools were analyzed on 32 arrays (4 arrays per pool), yielding 48 estimates of var(e_array_). Three of these estimates, each from pair-wise array comparisons involving the same array, were extreme outliers in both the normalized and raw dataset (see Figure 3). This array was determined faulty (see discussion) and removed from further analysis. For the remaining 45 estimates the mean var(e_array_) was 3.2 × 10^-4 ^(normalized) and 9.0 × 10^-4 ^(raw data), see Table 1. Unlike the data for the 1M-Single arrays, the 1M-Duo array data spanned two batches of genotyping, carried out at two different times. To look for batch effects the 1M-Duo data was also analyzed stratified by batch. The mean array variance was significantly different between batches for normalized data but not raw data (based on non-overlapping confidence intervals constructed assuming a normal distribution). Batch 1 (18 var(e_array_)) and batch 2 (27 var(e_array_)) had mean estimates of array variance of 4.2 × 10^-4 ^and 2.6 × 10^-4^, respectively. For the 660-Quad arrays, 7 pools were assayed using 72 arrays (6 or 12 arrays per pool), and mean array variance was 3.3 × 10^-4 ^for normalized data, and 2.7 × 10^-3 ^for raw data, see Table 1.

### Pooling Variance or var(e_pooling_): Type B and C comparisons

**Figure 4:**
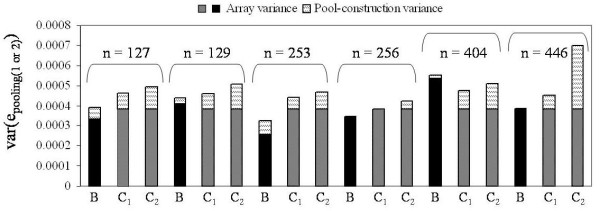
**Decomposition of pooling variance for Illumina 1M-Single arrays**. Stacked barplots showing the normalized pooling variance estimates, and the breakdown into array and to pool-construction variance for pools allelotyped on the Illumina 1M-Single array. Estimates derived from comparison of replicate pools are labeled "B". Estimates derived from comparison of non-identical pools are labeled "C_1_" and "C_2_" (specifying replicate pool). The portion of pooling variance attributed to pool-construction is indicated by hatched bars, and array variance by black or grey bars. Pool size is shown above the barplots.

We estimate pool-construction variance for 27 DNA pools, discussed in order by Illumina array type. Six pools were allelotyped on the 1M-Single array, and for each, pools were constructed in replicate and allelotyped by two arrays. This allowed us to calculate and compare pooling variance and pool-construction variance estimates as calculated using Type B and Type C comparison values. Figure 4 summarizes the var(e_pooling_) and var(e_construction_) estimates for those pools on the 1M-Single array. For normalized data var(e_pooling-1_) ranged from 3.2 × 10^-4 ^to 5.5 × 10^-4 ^and averaged 4.0 × 10^-4^. In comparison var(e_pooling-2_) ranged from 3.5 × 10^-4 ^to 7.0 × 10^-4 ^and averaged 4.8 × 10^-4^. Var(e_construction-1_) ranged from 0 to 6.7 × 10^-5 ^and had a mean of 2.9 × 10^-5 ^(where negative values have been set to zero). Thus, for these pools var(e_construction-1_) accounts for between 0 and 20%, or an average 7.5% of the pooling variance when using Type B derived values (see Additional File 2, **Table S2 **for all values). Var(e_construction-2_) ranged from 0 to 3.2 × 10^-4 ^and averaged 1.0 × 10^-4^; thus, pool-construction variance accounted for between zero and 46%, or an average 20% of the pooling variance using Type C derived values **(**Additional File 2, **Table S2**). There does not appear to be any correlation between pool size and pool-construction variance, see Figure 4.

Using raw data, estimates of var(e_pooling-1_) were approximately 8-fold higher than the normalized data. Estimates of var(e_construction-1_) tended to be higher as well, averaging ~20% of the pooling variance. Var(e_pooling-2_) estimates followed the same pattern, larger estimates of pooling variance and pool-construction variance (data not shown).

**Figure 5:**
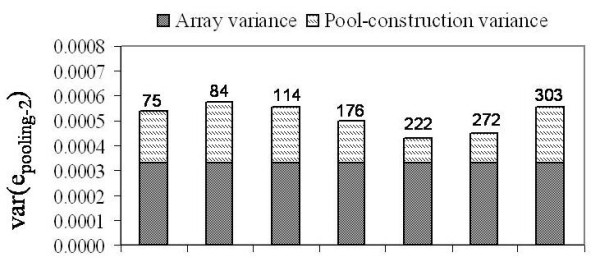
**Decomposition of pooling variance for Illumina 660-Quad arrays**. Stacked barplots showing the normalized pooling variance estimates, and the breakdown into array and to pool-construction variance for pools allelotyped on the Illumina 660-Quad array. All estimates are derived from comparison of non-identical pools, Type C. The portion of pooling variance attributed to pool-construction is indicated by hatched bars, the portion of pooling variance attribute to the array is indicated by grey bars. Pool size is indicated above each stacked bar.

Pools allelotyped on the 1M-Duo and 660-Quad arrays were not constructed twice; hence, for these we estimated pool-construction variance based on Type C comparisons only. Seven DNA pools were allelotyped on the 660-Quad array, two using six replicate arrays (396 estimates of var(e_pooling-2_) each), and five using twelve replicate arrays (720 estimates of var(e_pooling-2_) per pool. Figure 5 summarizes the var(e_pooling-2_) and var(e_construction-2_) estimates for these pools (normalized data). Var(e_pooling-2_) estimates ranged from 4.3 × 10^-4 ^to 5.7 × 10^-4^, and averaged 5.1 × 10^-4^; meanwhile, the var(e_construction-2_) estimates ranged from 1.0 × 10^-4 ^(23%) to 2.4 × 10^-4 ^(42%) and averaged 1.9 × 10^-4 ^(35%). These estimates of pooling variance are very similar to those seen for pools on the 1M-Single array; however, the estimates of pool-construction variance are higher (see Additional File 3, **Table S3 **for all values). For the raw data var(e_pooling-2_) estimates ranged from 2.6 × 10^-3 ^to 2.9 × 10^-3^, and averaged 2.7 × 10^-3^; meanwhile, the matched var(e_construction-2_) estimates ranged from 0 to 2.6 × 10^-4 ^(9%) and averaged 1.9 × 10^-4 ^(2%).

**Figure 6:**
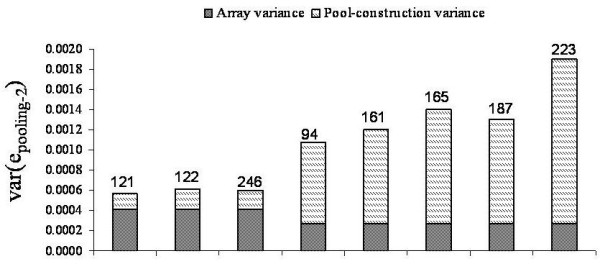
**Decomposition of pooling variance for Illumina 1M-Duo arrays**. Stacked barplots showing the normalized pooling variance estimates, and the breakdown into array and to pool-construction variance for pools allelotyped on the Illumina 1M-Duo array. All estimates are derived from comparison of non-identical pools, Type C. The portion of pooling variance attributed to pool-construction is indicated by hatched bars, the portion of pooling variance attribute to the array is indicated by grey bars. Pool size is indicated above each stacked bar.

1M-Duo arrays were analyzed separately by batch using batch-specific estimate of array variance for normalized data. The 1M-Duo batch 1 data contained three DNA pools, each allelotyped by four replicate arrays; therefore, each var(e_pooling-2_) estimate is the average of 32 pair-wise array comparisons. Figure 6 summarizes var(e_pooling-2_) and var(e_construction-2_) estimates for these pools (normalized data). Var(e_pooling-2_) was estimated at 5.6 × 10^-4^, 6.0 × 10^-4 ^and 6.1 × 10^-4^. The matched var(e_construction-2_) estimates were 1.5 × 10^-4^, 1.8 × 10^-4^, and 1.9 × 10^-4 ^, or 26%, 31%, and 32% of the pooling variance for pools sized 122, 246, and 121 (see Additional File 3, **Table S3 **for values). These values reflect those seen for pools on 660-Quad and 1M-Single arrays. In comparison, the 1M-Duo batch 2 data deviated dramatically. This batch contained 5 pools, each also alleloyped by four replicate arrays. For these var(e_pooling-2_) ranged from 1.8 × 10^-3 ^to 3.7 × 10^-3^, and averaged 2.6 × 10^-3^, and var(e_construction-2_) estimates ranging from 7.9 × 10^-4 ^(43%) to 2.7 × 10^-3 ^(72%) (see Additional File 3, **Table S3**). For these pools the estimates of pooling variance are nearly 2-3 fold higher than those of batch 1 but the array variance remained low at 2.4 × 10^-4^, leading to high estimates of pool-construction variance (see discussion). For raw data batch 1 & 2 were analyzed combined using all possible array comparisons and var(e_array_) = 9.0 × 10^-4^. Estimates of var(e_pooling-2_) ranged from 2.2 × 10^-3 ^to 5.4 × 10^-3 ^and averaged 3.4 × 10^-3^. Var(e_construction-2_) estimates averaged at 51% of the calculated var(e_pooling-2_).

### PoolingPlanner Example

**Figure 7:**
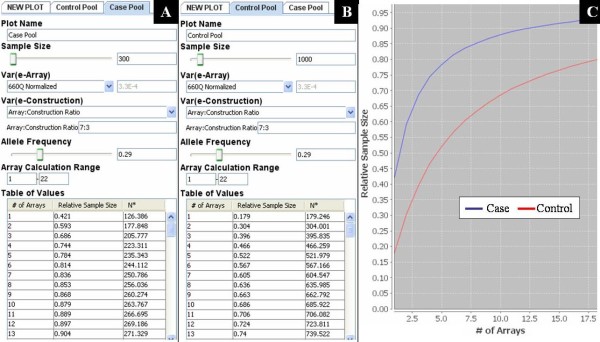
**PoolingPlanner**. (A) Control input and output panel for the case pool. (B) Control input and output panel for the control pool. (C) Corresponding plot of relative sample size versus the number if replicate arrays used in allelotyping the case (blue line) and control pool (red line).

To demonstrate how to use PoolingPlanner we consider a hypothetical scenario. A researcher has a collection of samples including 300 cases and 1000 controls and wants to conduct a pool-based GWAS. The researcher needs to decide how many arrays to use, and wants to construct power curves that take into consideration the power loss concomitant with this cost-efficient strategy. They plan on using Illumina's 660-Quad array and normalizing their data. PoolingPlanner is used to calculate the effective sample size of each DNA pool using four input values: 1) var(e_array_), 2) var(e_construction_), 3) pool size, and 4) allele frequency. Figure 7A shows the PoolingPlanner input panel for the case pool; Figure 7B the input panel for the control pool. PoolingPlanner will supply the var(e_array_) value as calculated based on our 660-Quad normalized data, 3.3 × 10^-4^, see Table 2. Alternatively, the user may specify a custom value. In this example we assume var(e_construction_) is 30% of the pooling variance, chosen to reflect values we observed. Var(e_construction_) is entered into PoolingPlanner by specifying "Array:Construction Ratio = 7:3", as seen in Figure 7A and 7B. An exact value for var(e_construction_) can also be entered (30% of 3.3 × 10^-4 ^would be 9.9 × 10^-5^). For allele frequency, by default PoolingPlanner uses HapMap CEU data (release 27) to set *p *to the average minor allele frequency (MAF) on the 1M-Single, 1M-Duo, or 660-Quad Illumina array. For the 1M-Single and 1M-Duo arrays *p *= 0.21 (>95% of SNPs had available HapMap data), and for the 660-Quad array *p *= 0.29 (87% of SNPs had available HapMap data). Estimates of *p *based on our pooled array data were similar (see Additional File 4, **Table S4**). In this example the average MAF is set to 0.29, but the user can enter any value between 0 and 0.5. Once these values are entered the program calculates the relative and effective sample size of each DNA pool for a range of replicate array values, and provides a corresponding table of values as seen in Figure 7A and 7B. A plot of relative sample size versus number of replicate arrays is also automatically generated. For a DNA pool containing 300 individuals (blue line in Figure 7C), an RSS of 80% is achieved with 6 arrays (N* is 244) while an RSS of 90% requires 13 arrays (N* is 271). In contrast, for a pool of 1000 individuals (red line in Figure 7C), an RSS of 80% is achieved with 19 arrays (N* is 806). This plot makes it easy to see at what point additional replicate arrays begin to yield diminishing returns in terms of increasing the effective sample size of a DNA pool.

**Table 2:** Impact of replicate arrays on effective sample size (N*) and minimum detectable odds ratio (MDOR) in pooling-GWAS.

Arrays per pool	Case pool (RSS, N*)	Control pool (RSS, N*)	MDOR at 80% (p = 0.29)	MDOR at 80% (p = 0.10)
**24**	0.95, 284	0.84, 837	1.33	1.51
**12**	0.90, 269	0.72, 720	1.35	1.54
**6**	0.81, 244	0.56, 562	1.38	1.58
**3**	0.69, 206	0.39, 391	1.44	1.70
**Individual Genotyping**	1, 300	1, 1000	1.32	1.49

This table compares the minimum detectable odds ratios (MDOR) at 80% power for a theoretical pooling experiment with 300 cases and 1000 controls, given a DNA-pooling strategy where 24, 12, 6, or 3 Illumina 660-Quad replicate arrays are used to allelotype each DNA pool (case and control). The equivalent individual genotyping experiment is given for reference. Relative sample size (RSS) and effective sample size (N*) are generated by PoolingPlanner assuming var(e_array_)= 3.3 × 10^-4^, var(e_construction_)= 9.9 × 10^-5^, and an average minor allele frequency of 0.29. MDOR at 80% power were calculated using Quanto [21] assuming an unmatched case-control design testing for gene-only effects using a log-additive model, where the incidence of the case phenotype is 0.02% and the risk allele, p, is set to 0.29 or 0.10.

**Figure 8:**
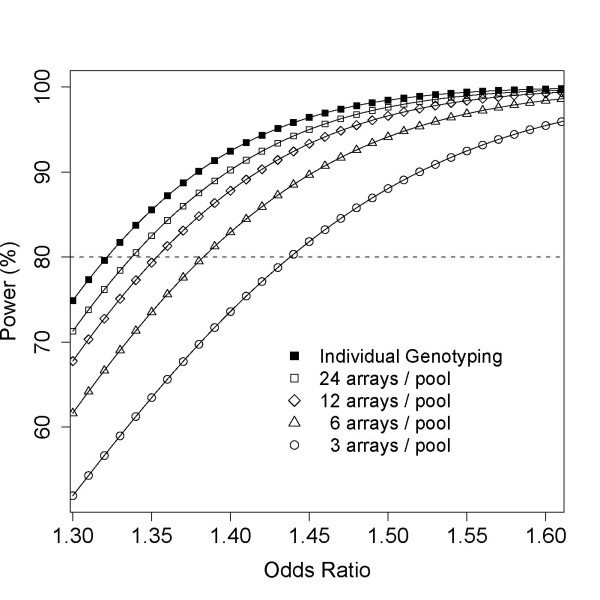
**Example use of PoolingPlanner**. Power curves for a theoretical pooling experiment with 300 cases and 1000 controls where 24, 12, 6, or 3 Illumina 660-Quad replicate arrays are used to allelotype the DNA pools. The equivalent individual genotyping experiment is given for reference. Effective sample size assuming 24, 12, 6, or 3 arrays was calculated using PoolingPlanner (see Table 2) and these values entered into Quanto [21] to obtain pool-adjusted estimates of power over a range of odds ratios. Calculations are based on an unmatched case-control design testing for gene-only effects using a log-additive model, where the incidence of the case phenotype is 0.02%, and the risk allele frequency (p**_risk_**) is 29% (and in complete linkage disequilibrium with a SNP on the array). A dashed line is draw to indicate the 80% power threshold.

To perform pooling-adjusted power calculations, a pool's effective sample size, output by PoolingPlanner, is entered into a power calculator. We have used Quanto [21] for this example. Assuming an unmatched case-control design testing for gene-only effects using a log-additive model, where the incidence of the case phenotype is 0.02%, and the risk allele frequency (p_risk_) is 29% (and in complete linkage disequilibrium with a SNP on the array), the power curves corresponding to a pooling experiment where 3, 6, 12, or 24 Illumina 660-Quad replicate arrays are used per pool is given in Figure 8. The power curve for individual genotyping is also plotted for reference. Table 2 accompanies this Figure 8 and gives the minimum detectable odds ratio (MDOR) at 80% power for each curve when p_risk _is 0.29, and for comparison, when p_risk _is 0.1. Assuming individual genotyping, the MDOR at 80% power would be 1.32 when p_risk _is 0.29. Using 24 arrays per pool this value rises incrementally to 1.33. Using 12, 6, or 3 arrays per pool, the MDOR's further increase to 1.35, 1.38, and 1.44, respectively. Only when 3 arrays are used per pool does the MDOR dramatically differ between pooling and individual genotyping. Marginal improvements in MDOR should be considered in light of increasing experimental cost, and the percent cost of a pooling GWAS relative to a conventional GWAS is given in Table 2 to highlight this difference. If arrays cost $250, the ability to detect an odds ratio of 1.38 with 80% power would cost $3,000 (6 arrays per pool), while the ability to detect an odds ratio of 1.33 would be $325,000 (individual genotyping). In many cases, particularly for phenotypes suggestive of moderate to large odds ratio, this difference in detectable odds ratios will not change of the overall outcome of the association study. In a pooling GWAS, as in conventional GWAS, for rarer risk alleles we have less power to detect associations, see the MDOR in Table 2 when p_risk _is 0.1. We note that as p_risk _gets smaller, the difference in the MDOR for a pooling versus individual genotyping experiment becomes more noticeable. For example, when 6 replicate arrays are used per pool and p_risk _is 0.29, the MDOR differs by 0.06 from individual genotyping, but this difference becomes 0.09 when p_risk _is 0.1. It is also worth noting in Table 2 that using the same number of replicate arrays on different sized DNA pools of very different RSS values. Contrary to what might be expected, the maximally powered pool-based experiment occurs when arrays are equally distributed amongst pools, regardless of differences in pool size and RSS, assuming the pool-construction variance is constant (see Additional File 5, Table S5 & Additional File 6, **Figure S1**). By conducting an analysis such as this a user can decide what power is forfeited by conducting a pool-based GWAS, and decide whether the approach makes practical sense in their situation.

## Discussion

In the first part of this study we set out to establish a range of experimentally observed values for array variance on Illumina's SNP-genotyping beadarrays. At the same time, we wanted to establish a range of values for pool construction variance. In the second part, we used these estimates to calculate the effective sample size of a DNA pool given a range of replicate array values, and provide an online tool to allow readers to do the same.

At the time of our analysis we were aware of only one report that estimated array variance (var(e_array_)= 1.1 × 10^-4 ^) for an Illumina HumanHap300 beadarray [18]. Illumina has since released higher density arrays (>1 million SNPs per array), and we wanted to determine if increased SNP density negatively impacted array variance. Overall, we found this was not the case. All of the Illumina array types examined here (660-Quad, 1M-Single, 1M-Duo) had very similar var(e_array_) estimates, centering around 3 × 10^-4 ^for our normalized data, which is largely in keeping with the HumanHap300 result [18]. We expect this result would extend to the HumanOmni1-Quad array, although it was not analyzed it here. We found that the normalization procedure we used reduced the array variance between 2-8-fold, and a newly reported normalization algorithm suggests that array variance can be reduced even further [24]. Reduced array variance should mean more precise estimates of allele frequency, which should further minimize the loss of power associated with using the DNA pooling strategy.

The Illumina arrays analyzed here yielded var(e_array_) estimates ~10-fold smaller than those of the Affymetrix *Hind*III 50K arrays (var(e_array_)= 1.26 × 10^-3^) analyzed by MacGregor [17]. A similar result was noted when Affymetrix arrays were compared to Illumina HumanHap300 arrays [18]. In part, this may be explained by differences in the manufacturing of the arrays. MacGregor et al. [18] report that pooling errors appear to be highly related to number of probes used to estimate SNP allele frequency. While 10 probe pairs are assigned to each SNP on the Affymetrix *Hind*III 50K arrays [18], on average 16-18 beads are used on the Illumina arrays. Further, on Illumina arrays beads are randomly dispersed on a slide [22], while on Affymetrix arrays probes are fixed in a given location, making the latter more susceptible to location-specific technical errors. As the array variance gets smaller (i.e. when using Illumina arrays), we expect the pool-construction variance to account for a greater proportion of the pooling variance.

Our estimates of var(e_construction_) spanned 27 DNA pools, ranging in size from 74 to 446 individual samples, allowing us to sample a range of possible pool construction variances. First, in contrast to a previous report [25], we did not observe a relationship between pool size and pool-construction variance. We did, however, observe batch effects. For the 1M-Duo arrays, which were processed in two batches on different dates, we observed very different estimates of pooling variance and pool-construction variance (see Figure 6). Most of our estimates of pool-construction variance were based on values from Type C comparisons, and for these var(e_construction_) usually fell between 20 and 40% of the pooling variance. When calculations were based on the comparison of replicate DNA pools (Type B comparisons, 1M-Single arrays only) our estimates were smaller, on average 7.5% of the pooling variance. There are several possible reasons for this. The adjustment for binomial sampling variance may not fully account for the variance arising from sampling, leaving variance that is then attributed to pool-construction in the Type C comparisons. As well, some estimates of pool-construction variance were negative, and these were set to zero, which would lead to overestimation of pool-construction variance. We conclude that relative to var(e_array_), var(e_construction._) is of less importance; however, our results suggest pool construction may account for more of the pooling variance than previously estimated [17]. MacGregor [17] attributed 12.5% of the pooling variance to pool-construction when using Affymetrix *Hind*III 50K arrays. On average we attribute 30% of pooling variance to pool construction when using Illumina arrays. This difference is what might be expected given the smaller var(e_array_) for Illumina arrays. Further reductions in array variance, for example, through improved normalization of array data, have the potential to further shift the proportion of an experiment's pooling variance that is attributed to pool-construction errors.

With respect to the design of pool-based experiments when using Illumina arrays, our partitioning of the pooling variance still suggests [17] that constructing fewer (large) pools while using more replicate arrays (i.e. target array variance), is the most effective way to reduce pooling variance and conduct the most efficient pool-based GWAS. Further, for an equivalent pool-based experiment using Affymetrix arrays in place of Illumina arrays, more array replicates will be needed (~10-fold more). As the proportion of array variance to pool construction variance approaches 50:50, strategies to reduce pool construction variance become more important.

For one of our experiments, 1M-Duo Batch 2, we observed unusually high estimates of pool-construction variance and low estimates of array variance (see Figure 6). In this experiment, pool replicates were allelotyped on the same physical array (which holds two samples). Subsequently, we noticed that the array variance for replicates on the same chip were much smaller than the variance for replicates on different chips. Overall, this led to the array variance being underestimated relative to the pooling variance, leaving more variance to be accounted for by pool construction. In addition, the between-chip variance for these arrays was much higher than observed in the 1M-Duo Batch 1 dataset, which lead to large estimates of pooling and pool-construction variance overall. Ultimately, this was traced back to unusually high red channel intensity on some arrays, despite normalization, which biased allele frequency estimates array-wide. Clearly this will influence any downstream association analysis, so in this case, our analysis of variance served to flag a serious problem in the array data. It also highlighted the need to randomize DNA pool replicates among arrays that carry more than one sample, and to randomize by location on the array, particularly in the case of the 660-Quad and HumanOmni1-Quad arrays, which carry four samples.

The differences between 1M-Duo Batch 1 and 2 data were significant for normalized data, but not raw data. On one hand, it may be that greater noise associated with the raw data prevented differences in array variance and pool construction variance from being significant. On the other, it is possible that the normalization procedure itself exacerbated technical artifacts only present on some arrays, leading to the observed differences in normalized data. This can occur if technical artefacts violate the assumptions of the normalization [26].

## Conclusions

We have provided empirical estimates of var(e_array_) and var(e_construction_) for a range of DNA pool sizes. We have also presented PoolingPlanner, a simple program to help translate these variances into their effect on sample size, information that can then be use in a power calculator to conduct pool-adjust calculations. PoolingPlanner may be helpful in quickly assessing theoretical best and worst-case scenarios for a DNA pooling GWAS. With this information the user can then make a more informed decision about how to carry out their pooling experiment to optimally balance cost with loss of power.

## Competing interests

The authors declare that they have no competing interests.

## Authors' contributions

MAE performed all statistical analysis and drafted the manuscript. KC developed and implemented the online tool PoolingPlanner. MR and ABW participated in study design, coordination, and manuscript drafting. All of the authors have read and approved the final manuscript.

## Pre-publication history

The pre-publication history for this paper can be accessed here:

http://www.biomedcentral.com/1755-8794/4/81/prepub

## Supplementary Material

Additional 1 **Additional Table S1**. Click here for file

Additional 2 **Additional Table S2**. Click here for file

Additional 3 **Additional Table S3**. Click here for file

Additional 4 **Additional Table S4**. Click here for file

Additional 5 **Additional Table S5**. Click here for file

Additional 6 **Additional Figure S1**. Click here for file
