# Supplementary material for: Estimates of array and pool-construction variance for planning efficient DNA-pooling genome wide association studies
Source: BMC Med Genomics. 2011 Nov 28;4:81. doi: 10.1186/1755-8794-4-81 (PMC3247851; doi:10.1186/1755-8794-4-81)
Supplement: Additional 1 — Additional Table S1. [file 1755-8794-4-81-S1.PDF]

Table S1: Details of the 27 DNA pools and 128 Illumina arrays used in the analysis of pooling variance

Pool No.	Pool name	Samples in DNA pool	Arrays per pool	Genotyping array	Genotyping batch/date	DNA extraction method
1	1-1M-Single 1	127	2	1M-Single	1-Apr-08	PAXgene Blood DNA Kit
2	1-1M-Single 2	127	2	1M-Single	1-Apr-08	
3	2-1M-Single 1	129	2	1M-Single	1-Apr-08	
4	2-1M-Single 2	129	2	1M-Single	1-Apr-08	
5	3-1M-Single 1	253	2	1M-Single	1-Apr-08	
6	3-1M-Single 2	253	2	1M-Single	1-Apr-08	
7	4-1M-Single 1	256	2	1M-Single	1-Apr-08	
8	4-1M-Single 2	256	2	1M-Single	1-Apr-08	
9	5-1M-Single 1	404	2	1M-Single	1-Apr-08	PureGene DNA Kit
10	5-1M-Single 2	404	2	1M-Single	1-Apr-08	
11	6-1M-Single 1	446	2	1M-Single	1-Apr-08	
12	6-1M-Single 2	446	2	1M-Single	1-Apr-08	
14	1-1M-Duo	121	4	1M-Duo	2-Oct-09	PAXgene Blood DNA Kit
15	2-1M-Duo	122	4	1M-Duo	2-Oct-09	
19	3-1M-Duo	246	4	1M-Duo	3-Oct-09	
13	4-1M-Duo	94	4	1M-Duo	2-Dec-09	Miller SA et al. (1988)
16	5-1M-Duo	161	4	1M-Duo	3-Dec-09	
17	6-1M-Duo	165	4	1M-Duo	3-Dec-09	
18	7-1M-Duo	187	4	1M-Duo	3-Dec-09	
20	8-1M-Duo	223	4	1M-Duo	3-Dec-09	
21	1-660-Quad	75	12	660-Quad	4-Mar-09	PureGene DNA Kit
22	2-660-Quad	84	12	660-Quad	4-Mar-09	
23	3-660-Quad	114	12	660-Quad	4-Mar-09	
24	4-660-Quad	176	6	660-Quad	4-Mar-09	
25	5-660-Quad	222	6	660-Quad	4-Mar-09	
26	6-660-Quad	272	12	660-Quad	4-Mar-09	
27	7-660-Quad	303	12	660-Quad	4-Mar-09	
