# Supplementary material for: Estimates of array and pool-construction variance for planning efficient DNA-pooling genome wide association studies
Source: BMC Med Genomics. 2011 Nov 28;4:81. doi: 10.1186/1755-8794-4-81 (PMC3247851; doi:10.1186/1755-8794-4-81)
Supplement: Additional 2 — Additional Table S2. [file 1755-8794-4-81-S2.PDF]

Table S2: Estimates of pooling variance, array variance, and pool-construction variance for DNA pools allelotyped on Illumina's 1M-Single array (normalized data)

Label[1]	var($e_{pooling(1\ or\ 2)}$)	var(e_{array})[2]	var($e_{construction(1\ or\ 2)}$)[3]	% C/P[4]
1-1M-Single, Type B	3.9E-04	3.3E-04	5.6E-05	14.5
2-1M-Single, Type B	4.4E-04	4.1E-04	2.9E-05	6.6
3-1M-Single, Type B	3.3E-04	2.6E-04	6.7E-05	20.6
4-1M-Single, Type B	3.2E-04	3.4E-04	0	0
5-1M-Single, Type B	5.5E-04	5.3E-04	1.9E-05	3.5
6-1M-Single, Type B	3.5E-04	3.9E-04	0	0
**AVERAGE**			**2.9E-05**	**7.5**
1-1M-Single 1, Type C_1	4.6E-04	3.8E-04	8.3E-05	18.5
1-1M-Single 2, Type C_2	4.9E-04	3.8E-04	1.1E-04	23.7
2-1M-Single 1, Type C_1	4.6E-04	3.8E-04	7.7E-05	17.5
2-1M-Single 2, Type C_2	5.1E-04	3.8E-04	1.3E-04	25.8
3-1M-Single 1, Type C_1	4.4E-04	3.8E-04	6.4E-05	15.1
3-1M-Single 2, Type C_2	4.7E-04	3.8E-04	8.6E-05	19.1
4-1M-Single 1, Type C_1	3.5E-04	3.8E-04	0	0
4-1M-Single 2, Type C_2	4.2E-04	3.8E-04	4.4E-05	11.1
5-1M-Single 1, Type C_1	4.7E-04	3.8E-04	9.3E-05	20.3
5-1M-Single 2, Type C_2	5.1E-04	3.8E-04	1.3E-04	25.9
6-1M-Single 1, Type C_1	4.5E-04	3.8E-04	7.1E-05	16.3
6-1M-Single 2, Type C_2	7.0E-04	3.8E-04	3.2E-04	46.1
**AVERAGE**			**1.0E-05**	**20.0**

[1] Variance estimates derived from comparison of replicate pools are labeled Type B. Variance estimates derived from comparison of non-identical pools are labeled Type C_1 or C_2. There are 6 unique pools but 12 pools in total because pools were constructed in replicate. The subscripts in C_1 and C_2 indicate the pool replicate.

[2] Type B var(e_{array}) is calculated from those arrays used to allelotype the unique pool specified. Type C var(e_{array}) is the average over all arrays over all pools (as in **Table 1**).

[3] Pool-construction variance is calculated as var($e_{construction(1\ or\ 2)}$) = var($e_{pooling(1\ or\ 2)}$) - var($e_{array}$), and negative values are set to zero.

[4] The percentage of pooling variance attributable to pool-construction variance.
