# Supplementary material for: Estimates of array and pool-construction variance for planning efficient DNA-pooling genome wide association studies
Source: BMC Med Genomics. 2011 Nov 28;4:81. doi: 10.1186/1755-8794-4-81 (PMC3247851; doi:10.1186/1755-8794-4-81)
Supplement: Additional 3 — Additional Table S3. [file 1755-8794-4-81-S3.PDF]

Table S3: Estimates of pooling variance, array variance, and pool-construction variance for pools allelotyped on Illumina's 1M-Duo and 660-Quad array (normalized data)

Label	$var(e_{pooling-2})$	$var(e_{array})$	$var(e_{construction-2})$[1]	% C/P[2]
1-660-Quad	5.41E-04	3.30E-04	2.10E-04	38.9
2-660-Quad	5.73E-04	3.30E-04	2.42E-04	42.3
3-660-Quad	5.59E-04	3.30E-04	2.29E-04	40.9
4-660-Quad	4.98E-04	3.30E-04	1.67E-04	33.6
5-660-Quad	4.30E-04	3.30E-04	9.98E-05	23.2
6-660-Quad	4.53E-04	3.30E-04	1.22E-04	27.0
7-660-Quad	5.59E-04	3.30E-04	2.29E-04	40.9
1-1M-Duo, Batch 1	5.63E-04	4.15E-04	1.48E-04	26.3
2-1M-Duo, Batch 1	6.07E-04	4.15E-04	1.92E-04	31.7
3-1M-Duo, Batch 1	5.98E-04	4.15E-04	1.83E-04	30.6
4-1M-Duo, Batch 2	1.07E-03	2.64E-04	8.10E-04	75.4
5-1M-Duo, Batch 2	1.20E-03	2.64E-04	9.34E-04	78.0
6-1M-Duo, Batch 2	1.40E-03	2.64E-04	1.14E-03	81.2
7-1M-Duo, Batch 2	1.30E-03	2.64E-04	1.03E-03	79.6
8-1M-Duo, Batch 2	1.90E-03	2.64E-04	1.64E-03	86.1

[1] Pool-construction variance is calculated as $var(e_{construction-2}) = var(e_{pooling-2}) - var(e_{array})$.

[2] Indicates the percentage of pooling variance attributable to pool-construction variance.
