# Supplementary material for: Estimates of array and pool-construction variance for planning efficient DNA-pooling genome wide association studies
Source: BMC Med Genomics. 2011 Nov 28;4:81. doi: 10.1186/1755-8794-4-81 (PMC3247851; doi:10.1186/1755-8794-4-81)
Supplement: Additional 4 — Additional Table S4. [file 1755-8794-4-81-S4.PDF]

Table S4: Comparison of average minor allele frequency on three Illumina arrays estimated using HapMap CEU data (release 27) and experimental pool-derived data

Pool Name	Pool Size	Average minor allele frequency	
		DNA pool	HapMap CEU
5-1M-Single	404	0.227	0.209
6-1M-Single	446	0.225	
1-660-Quad	75	0.260	0.288
6-660-Quad	272	0.262	
3-1M-Duo	246	0.249	0.208
5-1M-Duo	161	0.253	
