# Supplementary material for: Estimates of array and pool-construction variance for planning efficient DNA-pooling genome wide association studies
Source: BMC Med Genomics. 2011 Nov 28;4:81. doi: 10.1186/1755-8794-4-81 (PMC3247851; doi:10.1186/1755-8794-4-81)
Supplement: Additional 5 — Additional Table S5. [file 1755-8794-4-81-S5.PDF]

Table S5: Impact of unequal array allocation on effective sample size (N*) and minimum detectable odds ratio (MDOR) in a pooling genome wide association study

Arrays	Case pool		Control pool		MDOR at 80% (p=0.29)	MDOR at 80% (p=0.10)
Case : Control	RSS	N*	RSS	N*		
6 : 6	0.81	244	0.56	562	1.38	1.58
2 : 10	0.59	178	0.68	681	1.43	1.65
10 : 2	0.88	264	0.30	300		
4 : 8	0.74	223	0.63	631	1.39	1.60
8 : 4	0.85	256	0.46	461		

This table compares the minimum detectable odds ratios (MDOR) at 80% power for a theoretical pooling experiment with 300 cases and 1000 controls where 12 arrays are distributed differently between the case and control pools. Relative sample size (RSS) and effective sample size (N*) are generated by PoolingPlanner assuming vary(e_{array})=3.3x10^{-4}, var($e_{construction}$)=9.9x10^{-5}, and an average minor allele frequency of 0.29. MDOR at 80% power were calculated using Quanto (Gauderman & Morrison, 2006) assuming an unmatched case-control design testing for gene-only effects using a log-additive model, where the incidence of the case phenotype is 0.02% and the risk allele, p, is set to 0.29 or 0.10.
