# Supplementary material for: Estimates of array and pool-construction variance for planning efficient DNA-pooling genome wide association studies
Source: BMC Med Genomics. 2011 Nov 28;4:81. doi: 10.1186/1755-8794-4-81 (PMC3247851; doi:10.1186/1755-8794-4-81)

Figure S1 Legend

Figure S1: Power curves for a theoretical pooling experiment with 300 cases and 1000 controls where 12 arrays are distributed differently between the case and control pools. Effective sample size given the different pooling designs was calculated using PoolingPlanner (see **Additional File 5, Table S5**) and these values entered into Quanto to obtain pool-adjusted estimates of power over a range of odds ratios. Calculations are based on an unmatched case-control design testing for gene-only effects using a log-additive model, where the incidence of the case phenotype is 0.02%, and the risk allele frequency (p_{risk}) is 29% (and in complete linkage disequilibrium with a SNP on the array).

Figure S1

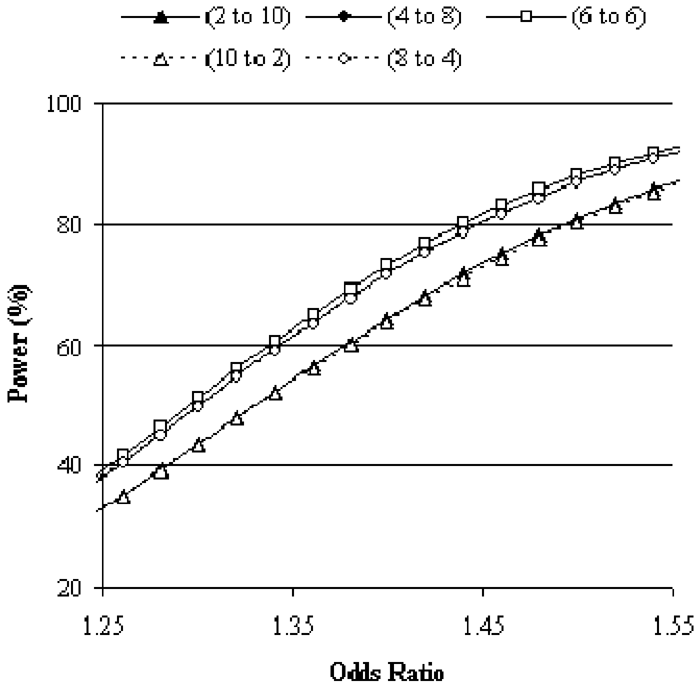

Supplement: Additional 6 — Additional Figure S1. [file 1755-8794-4-81-S6.PDF]
